# Perceptions of the “Fabric” – An exploratory study of a novel multi-purpose technology among women in Sub Saharan Africa

**DOI:** 10.1371/journal.pone.0204821

**Published:** 2018-10-31

**Authors:** Nicole D. Laborde, Jonah Leslie, Emily Krogstad, Neetha Morar, Prisca Mutero, Juliane Etima, Kim Woodrow, Ariane van der Straten

**Affiliations:** 1 Women’s Global Health Imperative, RTI International, San Francisco, CA, United States of America; 2 Department of Bioengineering, University of Washington, Seattle, WA, United States of America; 3 South Africa Medical Research Council, Durban, South Africa; 4 UZ-UCSF Collaborative Research Programme, Harare, Zimbabwe; 5 Makerere University-Johns Hopkins University Research Unit, Kampala, Uganda; 6 Center for AIDS Prevention Studies, Department of Medicine, University of California San Francisco, San Francisco, CA, United States of America; Beth Israel Deaconess Medical Center/Harvard Medical School, UNITED STATES

## Abstract

**Background:**

HIV and pregnancy prevention are dual health priorities for women, and particularly in sub-Saharan Africa. Drug-eluting fibers offer a dosage form that combines HIV prevention and contraception, but early understanding of end-user perspectives is critical to avoid misalignment between products being developed and preferred product attributes.

**Methods:**

Focus group discussions (FGDs) were conducted in South Africa, Uganda and Zimbabwe, among 55 women who had used vaginal products in previous trials. Participants were given the opportunity to feel a sample of electrospun nanofiber (the fabric), see how it dissolves, and give feedback on shape, size and other attributes. Women were also asked to compare the fabric to vaginal gel and film.

**Results:**

Three key themes regarding the acceptability of the fabric emerged: 1) look and feel of the product undissolved vs. undissolved, 2) expected effect on sex, and 3) convenience and ease of use. Upon being presented with the fabric, women were initially distrustful, seeing it as undesirable for vaginal insertion. Women generally approved of the product once they saw it dissolve. However, they stressed the importance of the product not interfering with sex by altering the vaginal environment. Women also reacted favorably to the perceived convenience of the fabric, particularly with regards to storage and transport, perceived ease of insertion and use, and dosing regimen.

**Conclusion:**

Multipurpose prevention technologies, and nanofibers in particular, should be developed with an eye to minimizing impact on sex while maximizing convenience, and presented in such a way as to emphasize non-abrasiveness and ease of dissolution.

## Introduction

Preventing HIV and unintended pregnancy are dual health priorities for women globally, and particularly in sub-Saharan Africa (SSA) where young women are infected with HIV at almost twice the rate of young men [1]. Suitable multi-purpose prevention technologies (MPTs) that combine HIV prevention and contraception in one delivery form may increase the acceptability and uptake of the combined product. For example, stigma related to HIV and the lack of trust implied in using an HIV prevention method with a partner are barriers to HIV prevention that could be mitigated by dovetailing HIV prevention with contraception [2, 3]. Various factors affect uptake and continuation contraceptive methods in sub-Saharan Africa, including barriers to access, partner influence, fear or experience of side effects, and inconvenience[3, 4]. Available MPTs (e.g., male and female condoms) have not been widely accepted as both men and women indicate that they interfere with sexual pleasure and are associated with mistrust and infidelity [5, 6].

Improved MPTs that respond to women’s preferences are urgently needed. Learning from adherence challenges in recent HIV prevention trials, acceptability and perceived usability are paramount to a product’s effectiveness and, ultimately, to its success. Recent studies have sought to better understand what product attributes and dosage forms women prefer for HIV prevention and MPT so that they can be considered for future product development[7–11]. One study reporting on various delivery mechanisms, including a daily oral pill, monthly injections, and a monthly vaginal ring found that acceptability of product attributes (eg. product look, ease of use and dosing regimen) contributed significantly to the rating of all products[9], and another found that preferred products varied by region, emphasizing the value of choice to accommodate diverse users[8]. Further, experts have called for integrating end-user perspectives at an earlier stage in design, though this approach remains rare[12, 13].

Drug-eluting nanofibers are a novel dosage form for intravaginal drug delivery that offer significant flexibility in product attributes to meet varied needs [14–19]. This dosage form is fabricated using electrospinning, a process in which electrostatic forces are applied to drug-containing polymer solutions to form micro- or nanoscale polymer fibers that are collected as soft, flexible fabric-like sheets, which can be processed into sheets, tubes, pessaries, or coatings. Electrospun nanofibers offer versatility in controlled drug release (quick vs. slow), geometry, mechanical stiffness, dissolution time, texture or “feel”, and thickness, among other properties. This research focuses on a form of electrospun nanofibers that are inserted vaginally and dissolve, referred to in the research study as the “fabric”[20].

To date, few prevention products have been developed with attention to properties that simultaneously promote both efficacy and user adherence and acceptability. Here we explore the perspectives of vaginal microbicide-experienced women in sub-Saharan Africa on a new potential vaginal dosage form for MPT, fabrics.

## Methods

This qualitative pilot “Fabric” Study was conducted in three countries in Africa at sites that were currently or had previously carried out clinical trials of microbicide vaginal gels: Durban, South Africa; Kampala, Uganda; and Harare, Zimbabwe. The study was carried out as a partnership between a bioengineering team developing the fabric and a qualitative research team experienced with end-user studies of vaginally inserted HIV prevention technologies. Each study site ran between 2 and 4 FGDs or small group conversations with 12–20 women per site for a total sample of 55 participants (Table 1). Researchers contacted women from the roster of previous microbicide gel study participants who had given written permission to be re-contacted for future studies and were between the ages of 18 and 49. These women were well qualified to identify salient end-user product attributes of the fabric, based on their prior exposure to vaginally inserted products for HIV prevention through their prior microbicide trial participation.

**Table 1:** Summary of data collection activities.

Site	Number of Women Contacted	Number of Women Who Agreed	Number of Participants who Attended FGDs	Number of FGDs
**MU-JHU, Uganda**	**26**	**26**	**20**	**2**
**MRC, South Africa**	**132**	**34**	**23**	3 (and 1 small group conversation)*
UZ-UCSF, Zimbabwe**	**14**	**14**	**12**	**2**
Total			**55**	**7**

* For their first FGD only 2 women showed up, so they held a small group conversation.

** Differing study timelines and time required for ethics approval resulted in variability in the number of FGD participants at each site, particularly for Zimbabwe.

Ethical approval for this study protocol was obtained from RTI's ethics committee and each of the local site's ethics committees prior to the study, including:

- Municipality of Chitungwiza, Department of Health Services
- Joint Research Ethics Committee for the University of Zimbabwe, College of Health Sciences and Parirenyatwa Group of Hospitals (JREC)
- Medical Research Council of Zimbabwe (MRCZ)
- Research Council of Zimbabwe (RCZ)

Uganda:

- Joint Clinical Research Center IRB, Kampala, Uganda
- Johns Hopkins Medicine, IRB

South Africa:

- South African Medical Research Council Ethics Committee

**Fig 1:**
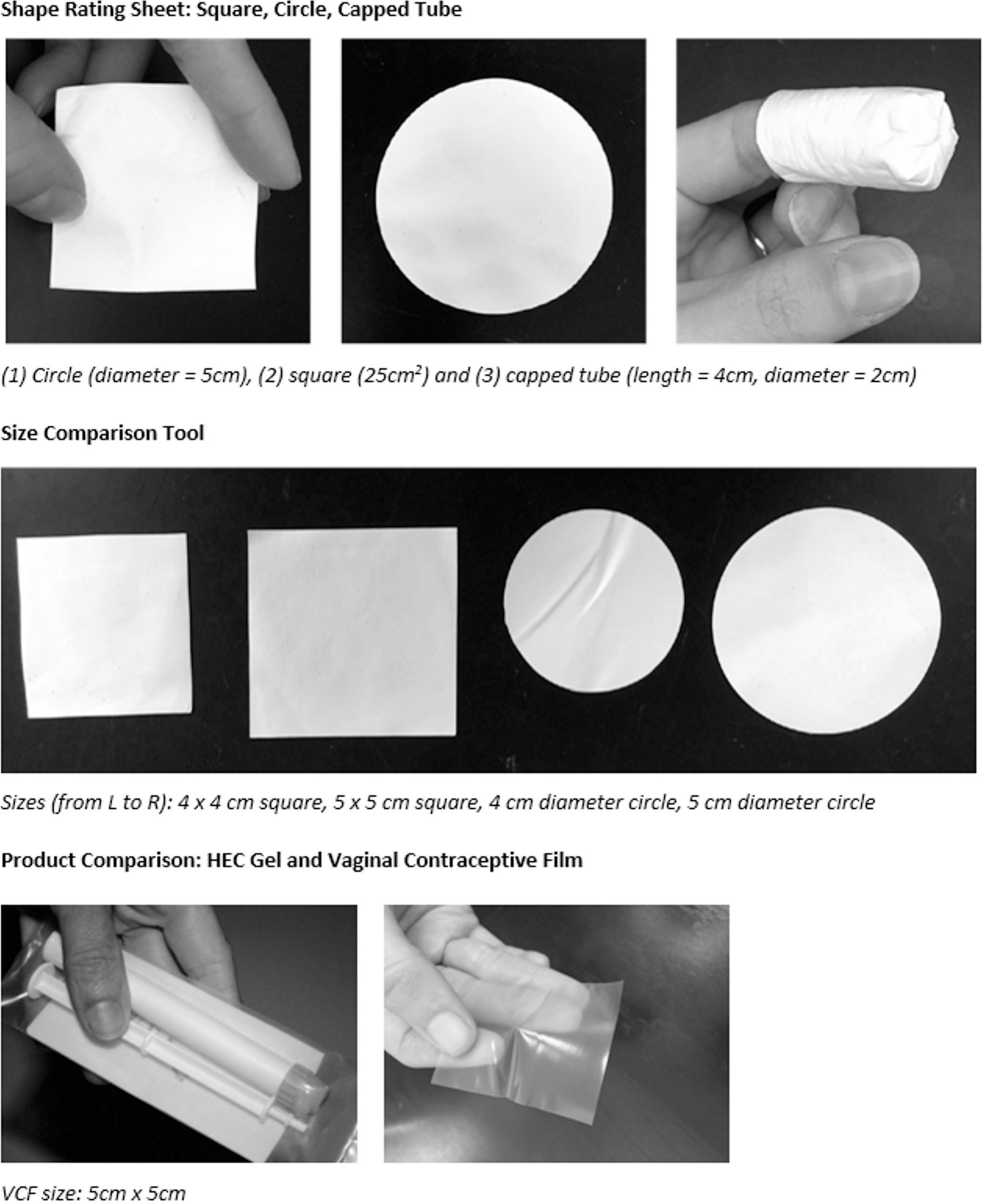
Shape rating sheet, size comparison tool, and product comparison.

FGDs were conducted by trained facilitators at each site in local languages. Members of the study team at each site had been involved in previous microbicide trials. Site study staff were trained in how to present the fabric during the FGD, such that participants would have similar experiences of observing and feeling the fabric both undissolved and dissolved. The FGDs ranged in length from 1 to 3 hours. Written informed consent was obtained from each participant prior to any other study procedures. Each participant was then asked to complete a short background and demographic questionnaire before the FGD began. Focus group questions and procedures are summarized in Table 2, and the main topics of discussion were interest in multipurpose technologies; experience with vaginal products; fabric physical attributes; fabric dissolution and dosing regimen; acceptability of fabric; and acceptability of gel and vaginal contraceptive film (VCF) as a means of comparison. In the FGDs, participants were given a chance to see and touch three shapes of the fabric (square, circle, and capped tube; Fig 1), and filled out a Shape Rating Form indicating their preference for shape. Participants also observed how the fabric dissolved in a small amount of water and felt the dissolved sample. No fabric prototypes were vaginally inserted, and none of the fabric prototypes contained any active ingredients. The characteristics of the fabric were compared with those of a placebo vaginal gel (hydroxyethylcellulose or HEC placebo gel) (Starpharma Holdings Limited, Melbourne, Australia) and Vaginal Contraceptive Film (VCF) (Apothecus Pharmaceutical Corp, Oyster Bay, NY), all of which were handled only (not vaginally inserted). The vaginal film was included because it is the most similar existing dosage form to the fabric: both are solid-state dosage forms that could be applied pericoitally and dissolve in the vagina. Comparing with the film enabled facilitators to probe differences in attribute preferences for solid-state dosage forms (e.g., differing opacity, texture, geometry for film v. fabric).

**Table 2:** Focus group guide topics and product demonstrations.

Major discussion topics	Subtopics
Interest in multipurpose prevention technologies	• Advantages or disadvantages • Community reaction • Partner’s reaction and interest
Experience with vaginal products	• Purposes of vaginal products • Vaginal washing • Vaginal HIV prevention products • Interference between vaginal practices and a vaginal MPT product
Prototype demonstration: geometry	• Participants given 3 fabric geometries–circle, square, capped tube—to feel in their hands (undissolved) • Pelvic model used to demonstrate insertion*
Fabric physical attributes (geometry and insertion)	• Shape preferences • Perceived ease and difficulty of insertion • Storage, transportability, privacy issues • Size • Thickness, texture, edges, strength, etc. • Applicator preference • Perceived likes/dislikes by others (husband/ partners, community members)
Prototype demonstration: dissolution	• Facilitators added a small amount (~1 ml) of water to circle fabric prototype in plastic bag so that participants could observe dissolution • Participants felt dissolved prototype in plastic bag
Fabric dissolution and dosing regimen	• Frequency of insertion • Dissolution time • Feel of product once dissolved
Acceptability of fabric	• Community reaction • Male partner reaction • Suggested names
Acceptability of gel	• Experience with gel • Comparison of gel to fabric
Acceptability of vaginal contraceptive film (VCF)	• Reaction to VCF** • Comparison of VCF to fabric
Opinions and preference on three potential MPTs	• Comparison between gel, VCF, fabric

*Pelvic model demonstration of insertion only used in some FGDs.

**VCF was also dissolved in small amount of water in some FGDs.

FGDs were translated and transcribed from the local language into English, and transcripts underwent a quality check for clarity before being uploaded into Dedoose qualitative software[21]. Data analysis began with textual data coding to summarize and organize the data. A code book was developed by two qualitative analysts at RTI starting with topics from the focus group guides. Additional codes were added as additional themes and areas of interest emerged from the focus group transcripts. Both analysts read all transcripts prior to coding, and coding was initiated with double-coding to ensure inter-coder reliability. Coding queries were run for use in retrieving text on given topics to assist with summarizing the FGD data, and summary memos were written on coding queries of principal attributes (e.g., fabric geometry, method of insertion, fabric size, onset and duration of dosing, fabric dissolution, physical appearance of fabric). The coded data set and summary memos were used to write an initial report for the product development team on end-user preferences for key product attributes such as geometry, texture, and size, attributes that could be modified during design. Through this process, we identified three themes that spanned a variety of. These themes were refined through team discussion between the bioengineering team and qualitative research team. Pseudonyms are used in presenting the data to protect the identity of participants.

## Results

### Participant demographics

A total of 55 women participated in the study: 12 from Harare, Zimbabwe; 23 from Durban, South Africa; and 20 from Kampala, Uganda. Participants had a median age of 30 and had a median of 2 children (Table 3). Of the participants, 95% were married or had a primary sex partner. Of these women, 43% were currently living with their partners and 94% had vaginal sex with their primary partner in the last three months. All study participants had previously used the placebo (HEC) or active tenofovir vaginal gel in other research studies.

**Table 3:** Background and demographic characteristics.

	*All*	*Uganda*	*South Africa*	*Zimbabwe*
***Age*** *median (mean, min-max) [N]*	30 (31.8, 25–44) [55]	34 (33.5, 25–42) [20]	28 (30.3, 25–44) [23]	31 (31.8, 26–39) [12]
***Number of Children*** *median (mean, min-max) [N]*	2 (2.3, 0–5) [55]	3 (3.1, 0–5) [20]	1 (1.6, 1–4) [23]	2 (2.1, 1–4) [12]
***Education***				
*Some/complete Primary School*	13 (24%)	12 (60%)	1 (4%)	-
*Some/complete secondary school*	39 (71%)	7 (35%)	20 (87%)	12 (100%)
*Attend College or University*	3 (5%)	1 (5%)	2 (9%)	-
***Religion***				
*Christian*	47 (85%)	17 (85%)	18 (78%)	12 (100%)
*Muslim*	3 (5%)	3 (15%)	-	-
*Other*	5 (9%)	-	5 (22%)	-
Has partner*	53 (96%)	19 (95%)	23 (100%)	11 (92%)
Currently living with partner*	23 (43%)	11 (58%)	3 (13%)	9 (82%)
***Transactional sex in the past year***	13 (24%)	12 (60%)	1 (4%)	-

*Partner includes being married or having a primary partner.–indicates zero value.

### Interest in MPTs

At the start of the focus groups, the idea of multipurpose technology was explored generally for women’s opinions and preferences. Women generally reacted positively to the idea of multipurpose technologies, highlighting the utility of having a single product to prevent both HIV and pregnancy. Several described various risk factors for acquiring HIV (e.g., multiple sex partners) that motivated their desire for an MPT product. As Sarah (Uganda, age 33) indicated, “I am so happy for that idea of having a single product because I… I do not have only one partner, I have other men as side dish [side partners] but in case that product is available it would help to protect my life so that I can be able to look after my children well without getting infected.”

Women also postulated that male partners would approve of an MPT, both because it would help prevent them from contracting HIV if their female partners contracted it somewhere else, and because they would no longer have to wear condoms. Nikita (Uganda, age 31) explained that, “some men say that a condom is too tight, it fails them to ejaculate. Doing away with the condom will be a flesh to flesh experience, thus we will enjoy the pleasure without the worry of pregnancy or HIV—that is if it works out.”

A few concerns were raised about potential negative aspects of an MPT. Foremost, some women and/or their partners wanted to have children: “What I see as a bad side of it is; in case you had a partner and you would like to get pregnant but your partner is HIV positive … If they could design one which can prevent HIV only and allow those who want to get pregnant go ahead and give birth.” (Terisa, Uganda, age 29)

Some women also pointed out the increased risk of side effects, as an MPT would be functioning for multiple indications and thus would be affecting the body in multiple ways. A minor theme around religious prohibition of the use of contraception emerged as well. A few participants raised this as a potential barrier in their communities.

Participants had mixed feelings about whether their partners would approve of a vaginally inserted prevention method at all, even beyond its effect on sex. Some felt that their partners’ primary objections would be about the contraceptive indication rather than the HIV prevention indication. Others said their partners simply do not like when women are inserting things vaginally, potentially for safety concerns or from a general dislike of vaginally inserted products.

Part of the concern for using a vaginally inserted product related specifically to menses. Some women such as Zamokuhle (South African, age 32) didn’t see a problem, “Because it is not going to stop your menses.” Others, however, found the idea of inserting a product during active blood flow distasteful, or thought if they are feeling unwell during menses, they might not want to use it. Some thought it would not stick or that it would come out with the blood and therefore be less effective. Naira (Uganda, age 36) summed this up: “I wouldn’t want to open that place when I am in my menstrual periods. I think even another person would not want to open there and insert it. Remember it is a period of blood flowing, it might not work properly.” Some women stated they did not have sex during their menses and therefore did not need protection, while others said their husbands didn’t mind having sex if they are in their periods so they should still seek protection. Several times, women mentioned that a longer-lasting product (weekly or greater) could enable insertion outside of their period.

### Reaction to the fabric

When first presented with the fabric, women were initially unsure of how it would work and were confused about the idea of a dissolving “fabric”. Once they saw it dissolve, they were generally very pleased. Of the 3 different shaped fabric samples (circle, square and capped tube), they liked the capped tube shape best, followed by the circle, and the square least, primarily because they felt the first two would be easiest to insert and they did not like the hard edges of the square (Table 4). Women preferred the smaller size of the fabric prototypes for the most part, again because of perceived ease of insertion. When asked about duration of action, women said they would prefer a product that would last for a week or longer over a shorter acting product. They also preferred the product to dissolve as quickly as possible to get immediate protection and to avoid detection by male partners.

Three key themes regarding the potential acceptability of the fabric emerged from our analysis of the Focus groups (FGDs): 1) look and feel of the product undissolved vs. undissolved, 2) expected effect on sex, and 3) convenience and ease of use. Focus group facilitators asked women to compare the fabric to gel and to the vaginal contraceptive film (VCF). The comparison offered a means of gaining more specific insights into desirable and undesirable attributes.

**Table 4:** Shape preferences.

	All	Uganda	South Africa	Zimbabwe
**Shape liked most**				
Square	6 (11%)	1 (5%)	5 (22%)	-
Circle	8 (15%)	6 (30%)	-	2 (17%)
Capped tube	41 (75%)	13 (65%)	18 (78%)	10 (83%)
**Shape liked least**				
Square	40 (74%)	14 (70%)	16 (73%)	10 (83%)
Circle	5 (9%)	1 (5%)	2 (9%)	2 (17%)
Capped tube	9 (17%)	5 (25%)	4 (18%)	-

### Attributes of the products undissolved vs. dissolved

When initially presented with the undissolved fabric, women were unsure of how it would work. The fact that it was called “fabric” (often translated in local languages as cloth) in the study was at times confusing, such that the product did not look like what they imagined, and it was difficult to imagine cloth/fabric dissolving.

When they first saw and handled the fabric prototype, women often likened it to paper, and stated they would be uncomfortable with inserting a piece of paper in their vaginas. Zondile (South Africa, age 28) commented, “It is difficult and scary my sister …Vagina is a very sensitive part, I always make sure that soap does not get into my vagina—how much more for a paper that will have to go to my vagina.”

Softness was an important quality for women at all three sites, and some participants worried that the undissolved fabric would scratch or be too hard. As Hellen (Uganda, age 38) indicated, “[W]hether square or circle or tin [capped tube], it should be softened …I think they will be able to make it softer than it is now.”

Other women, however, commented on the product’s softness as a determining factor in how much they like it. Zamokuhle (South Africa, age 32) said, “I don’t see any problems because it is soft. It would have been difficult if it was like steel, and you are required to keep the steel inside? Now you will keep something that is a fabric, it will be kept nicely.”

After seeing it dissolve, many women’s attitude improved toward the expected texture of the fabric after insertion. For example, Nami (South Africa, age 25 explained, “I think it’s better [after seeing it dissolve] because if it’s going to be inserted like a circle or square I thought it might hurt you and move to the womb. But now I think it is much better.” Women were generally very surprised and excited to see the product dissolve in water and were more confident about the product after seeing how it worked.

In most of the FGDs, participants similarly did not like the undissolved feel of the VCF, which they indicated looked like plastic. Asanda (South Africa, age 25) shared a common sentiment, “It’s like they made a mistake by designing this thing [VCF] into a plastic form, I think I will need to go through an operation in order to remove this thing, does it really dissolve?”

In some of the FGDs where the VCF was also shown to dissolve, women were better able to understand the nature and performance of the product, and their interest in the product increased. Nevertheless, the perception of the VCF as plastic remained a barrier to its potential acceptance.

### Expected effect on sex

Women likened the dissolved fabric to natural fluids, indicating that it looked like vaginal discharge or like semen, which was positively perceived. In comparison with the gel, women thought the fabric would not make sex as slippery because of the difference in volume between the two products, as Getu (Uganda, age 36) expressed. “I think the piece of cloth [fabric] will work for me because it won’t be as much as the vaginal gel. The vaginal gel used to be so much but this piece of cloth [fabric]; I feel if I inserted it, it will make sex good, he [partner] will not feel so much fluid the way he used [to] with the vaginal gel.”

The balance between being wet or slippery and being dry or gluey seemed to be important for Paida (Zimbabwe, age, 26), as for other women in the study. “We do not want one that is too slippery so that [sex] will not be felt. We do not want one like that. We do not want one which is too slippery.” Terisa (Uganda, age 29) joked that women would be begging for the product: “When you are dry and the man is also dry it might cause some wounds and mix the blood. So, if they increase that starch [sperm-like content], you will not plead with us to use them but rather us to plead with you to make more of them.”

The women’s primary concern regarding their partners was how the product would impact sex. They stressed the importance that the fabric not change the way their vaginas feel to their sexual partner, and that it not make them too dry or too wet, which would negatively impact the pleasure they and their partners felt. They generally stated that they thought the current fabric prototypes would either not change the vaginal environment for sex or would change it positively by helping with lubrication.

The expected impact of the fabric on sex influenced their opinions about how long it should take to dissolve and also how long the protection from the product should last. For the most part, women wanted the fabric to dissolve quickly so that it did not interfere with the timing of sex. There was some concern that the capped tube shape might not dissolve as quickly because it appeared thicker due to folds in the fabric. In regard to duration of protection and impact on sexual pleasure, participants indicated that if the product made sex pleasurable, women might prefer to use it more frequently. In contrast, if it is not pleasurable, they would want to use it less frequently (or not precoitally). Participants often described sexual pleasure as connected to the amount of lubrication, vaginal tightness, or heat, though women were vague on specific conditions or comparisons. Mary explained (Zimbabwe, age 39), “It depends with whether you won’t get watery down there [vagina] after you have inserted it …Because it can become ‘loose, loose’… If it becomes ‘loose loose’, that [inserting] once a month will be fine…But if it does not cause that, you can say once a week. If it is too wet [laughs], the father [husband] will not like it.”

In sum, the effect of the dissolved fabric on sex was an important consideration for women in these FGDs in determining overall willingness to use the product, influencing their preferences for specific characteristics such as dissolution time, shape, and duration of protection.

### Convenience and ease of use

The third theme that emerged was the perceived convenience of using the fabric. This applied to storage and transport, perceived ease of insertion and use, and dosing regimen.

Women liked that the fabric was small and easily transported, especially compared to the vaginal applicator and gel. One participant even mentioned it could be carried in her bra, or in Madhuve’s (Zimbabwe, age 27) case, her pocket, “It is good because it’s different from gel. The gels were bulky but this one is better, you can even put it in the pocket.” Women felt it was discreet and no one would pay attention to it.

Convenience was one of the primary drivers in determining how often women would like to use a product and how long protection should last. Facilitators asked women if they would be interested in a product that was used before sex, daily or weekly. Women generally wanted a longer-acting product. *“*I want it to be monthly. We work, you come back very tired, you just bathe you won’t remember inserting it, in the morning you wake up very quickly, the kids… then you have to go to work. Let it be monthly.” (Rania, Uganda, age 25) Some asked for a product that would last up to three months.

Perceived ease of use was another important attribute that informed acceptability. In most of the groups, women preferred the fabric to the gel. Women had negative experiences with gels during previous trials with the gel leaking out and feeling wet, and they did not think this would be the case with the fabric. Some did not like having to wear a panty liner (provided as part of standard procedures in the gel studies) when using the gel and felt this would not be needed with the fabric. Women also said that unlike the gel, the fabric would not make them “cold” because they would not be inserting a liquid.

Perceived ease of insertion was a major determinant in shape preferences. The shape rating survey showed that 75% of participants preferred the capped tube (Table 4), and many cited the tube’s apparent ease of insertion as the reason for their choice. The capped tube did not require wrapping around the finger for insertion and would be unlikely to come unfolded before it was properly placed in the vagina. Others felt the capped tube might stick on the finger and thus preferred the circle. Also, when comparing fabric sizes, most women preferred the smaller sizes because they thought it would be easier to insert and it would dissolve more quickly. “Why I have liked the small size is because it will be easy for me to insert it. While inserting it, I won’t be sluggish like a cat tracking down a rat. It will be easy for me to use.” (Nanteza, Uganda, age 41)

Overall, convenience and ease of use were important factors for women, as evidenced by their preferences for a small fabric size and discreet packaging, as well as their wanting to insert a product less frequently and more easily.

## Discussion

In this qualitative study among 55 women in sub-Saharan Africa, women indicated their preferences for specific characteristics of a novel MPT formulation, the fabric, such as shape, size, dosing regimen, and duration. Women also compared the potential acceptability of the fabric, to the vaginal gel and VCF. Participants were initially unsure about the look and feel of the fabric before it had been dissolved in water, expressing concerns about how it would dissolve and if it was soft enough to insert. These concerns decreased once participants saw how the fabric dissolved. They expected that once dissolved the fabric would not interfere with sex and stated that the dissolved product looked like a natural fluid. Women wanted a product that would be discreet and convenient, and the fabric met both criteria. Overall women were very interested in the idea of an MPT and wanted a product that is infrequently inserted and that will not raise concerns with their partners.

The finding here that women are uncomfortable with products that are not familiar to them resonates with findings from other qualitative studies on formulations for HIV prevention [22–24]. Because the fabric is a novel formulation that does not resemble other vaginal products, many women did not initially understand how it was going to work. Its paper- or cloth- like appearance suggested to them that it would not dissolve. Once they were able to see how it dissolved in water, they were more comfortable with it and recommended that such a demonstration would make the product more attractive to other women.

The potential impact of a product on sex emerged as an important theme. Previous microbicide trials have found that the impact of the product (e.g. vaginal gel or vaginal ring) on sex was a primary concern for women in using it and influenced women’s willingness or ability to use the product [3, 25, 26]. Similar to women in vaginal gel and vaginal ring studies, many women in this study expressed a desire for a discreet product that would not be noticeable during sex; i.e. would not cause too much wetness, dryness, or leakage during use, and resembled natural fluids like vaginal fluid or semen once dissolved. On the other hand, participants indicated that if a product could enhance sex, for instance by slightly increasing lubrication, tightening the vagina, or creating heat, they would be more willing to use it.

Convenience of an MPT was important to women in the Fabric study, as women contrasted to their previous negative experiences with the vaginal gel of gel leakage, feeling wet, and feeling over-lubricated during sex. Further, women complained that the box containing the gel and applicator was bulky to carry around and that is was difficult to dispose of the applicator discreetly [3]. In a previous microbicide trial participants also indicated that they forgot to take daily doses of products. Our findings in the Fabric study resonate with these findings, in that many would prefer to have protection that lasts longer, can be used discreetly, and is less likely to be forgotten.

The fabric is one of several MPT products, including vaginal ring, film, gel, and oral tablets, being developed to appeal to women with different needs and concerns [27]. End-user studies that aid in developing products with characteristics desired by target populations are critical [28]. The fabric is a new dosage form for vaginal drug delivery that feels different, looks different, and could come in a different shape relative to other vaginal products like vaginal gels and vaginal films; thus, these unique features of the fabric may be preferable to some women.

Limitations of the study include the small sample size, which did not allow for comparisons based on site or demographics. However, the study was intended to be a pilot study that provides a diversity of insights into women’s preferences for a vaginally inserted MPT product, focused on obtaining perspectives on a novel dosage form of a fabric. The educational level of women differed between sites and could have impacted their reactions to products. However, the fact that all women were experienced with a vaginal gel and were HIV prevention trial-literate provided a strong basis for them to offer their opinions on this product in development. Additionally, since the fabric was presented first during the FGDs and discussed much more extensively than the gel or film, participants may have been biased to react more favorably toward the fabric dosage form. Although women did not insert the products into their vaginas to experience how it would feel, the relevance of participants’ feedback was enhanced by the fact we selected women who had previous experience with vaginally inserted products as a basis for comparison.

Our purpose here was to gather end-user feedback at a relatively early stage in product development, when design changes can be more easily iterated on and implemented, compared to the clinical stage. Future acceptability studies in which participants can vaginally insert fabric products with varying characteristics would be insightful as this dosage form is further developed.

## Conclusion

Women experienced in the use of vaginal products in three sub-Saharan African countries showed interest in MPTs and indicated that an MPT product might help to overcome some issues that HIV prevention in isolation presents. Specifically, they stated that the fabric would be of interest as an MPT because they did not expect it to interfere with sexual experiences and thought it would be discreet and convenient to use. Novel dosage forms will require adequate user education and familiarization to encourage uptake.

Though individual preferences for these three vaginal dosage forms differed, most women in this study preferred solid-state dosage form of the fabric to the semi-solid dosage form of the gel as it was perceived to be less messy. Other preferences that emerged for vaginal dosage forms in this study were for soft texture (no sharp edges), convenient size and packaging, and longer-lasting duration (~1 week or more). Product developers of these dosage forms and other novel vaginal drug delivery products should consider how to prioritize such preferences during design. For example, for the fabric specifically, some women in this study said they would prefer if the protection from the fabric could last longer after a single dose than 24 hours, perhaps a week or longer. Other features of the fabric that women preferred such as a capped tube geometry for easier insertion, a softer texture, and a post-dissolution viscosity and appearance similar to vaginal fluid / semen are being considered as preclinical development of the fabric continues.
